# N-glycosylation converts non-glycoproteins into mannose receptor ligands and reveals antigen-specific T cell responses *in vivo*

**DOI:** 10.18632/oncotarget.14314

**Published:** 2016-12-28

**Authors:** Christoph Kreer, Janina M Kuepper, Matthias Zehner, Thomas Quast, Waldemar Kolanus, Beatrix Schumak, Sven Burgdorf

**Affiliations:** ^1^ Life and Medical Sciences (LIMES) Institute, University of Bonn, 53115 Bonn, Germany; ^2^ Institute for Medical Microbiology, Immunology and Parasitology (IMMIP), University Hospital Bonn, 53105 Bonn, Germany

**Keywords:** antigen presentation, Komagataella phaffii, receptor-mediated endocytosis, T cell activation, cross-presentation

## Abstract

N-glycosylation is generally accepted to enhance the immunogenicity of antigens because of two main reasons. First, the attachment of glycans enables recognition by endocytic receptors like the mannose receptor (MR) and hence increased uptake by dendritic cells (DCs). Second, foreign glycans are postulated to be immunostimulatory and their recognition could induce DC activation. However, a direct comparison between the immunogenicity of N-glycosylated vs. de-glycosylated proteins *in vivo* and a direct effect of N-glycosylated antigens on the intrinsic capacity of DCs to activate T cells have not been assessed so far.

To analyze whether enforced N-glycosylation is a suited strategy to enhance the immunogenicity of non-glycosylated antigens for vaccination studies, we targeted non-glycoproteins towards the MR by introduction of artificial N-glycosylation using the methylotrophic yeast *Komagataella phaffii* (previously termed *Pichia pastoris*). We could demonstrate that the introduction of a single N-X-S/T motif was sufficient for efficient MR-binding and internalization. However, addition of N-glycosylated proteins neither influenced DC maturation nor their general capacity to activate T cells, pointing out that enforced N-glycosylation does not increase the immunogenicity of the antigen *per se*. Additionally, increased antigen-specific cytotoxic T cell responses *in vivo* after injection of N-glycosylated compared to de-glycosylated proteins were observed but this effect strongly depended on the epitope tested. A beneficial effect of N-glycosylation on antibody production could not be detected, which might be due to MR-cross-linking on DCs and to concomitant differences in IL-6 production by CD4^+^ T cells.

These observations point out that the effect of N-glycosylation on antigen immunogenicity can vary between different antigens and therefore might have important implications for the development of vaccines using *K. phaffii*.

## INTRODUCTION

Dendritic cells (DCs) play an essential role in the initiation of adaptive immune responses. They are specialized in monitoring the immune state of peripheral tissues by taking up large amounts of extracellular antigens. Upon antigen recognition, DCs mature and migrate towards the draining lymph node, where they can activate antigen-specific T cells. Therefore, DCs process the internalized antigens and load antigen-derived peptides onto major histocompatibility complex (MHC) molecules. Presentation of antigens on MHC II molecules can lead to the activation of CD4^+^ T helper cells, whereas the presentation of exogenous antigens onto MHC I molecules, a process termed cross-presentation, can activate antigen-specific CD8^+^ cytotoxic T cells.

In a previous study, we could demonstrate that the decision whether internalized antigens are presented onto MHC I or MHC II molecules is mainly determined by the mechanism of endocytosis [1]. Antigens internalized by scavenger receptor-mediated endocytosis or by fluid phase pinocytosis were rapidly targeted towards lysosomes, where they were processed by lysosomal proteases for presentation onto MHC II. However, if the same antigen was internalized by the same DC via the mannose receptor (MR), it was targeted towards a distinct pool of endosomes and processed for cross-presentation [1, 2].

The MR is a member of the C-type lectin receptor family expressed on a variety of DCs, macrophages and endothelial cells [3]. It consists of an N-terminal cysteine-rich domain (CR), a collagen-binding fibronectin type II domain (FN II), eight C-type lectin-like domains (CTLDs) a transmembrane region and a short cytosolic region. The CTLD4 of the MR is sufficient for binding to the monosaccharides mannose, fucose or N-acetyl-glucosamine, but at least three CTLDs (CTLD4, 5 and 7) are necessary for efficient binding and internalization of multivalent ligands [4].

The endocytic capacities of receptors like the MR made them an interesting target in vaccination studies aimed at the induction of a strong T cell response, e.g. against viruses or in tumor vaccination studies. Therefore, in various approaches, antigens were directly targeted towards such receptors by conjugation to specific antibodies or sugar moieties [5, 6, 2, 7]. One approach to produce high amounts of glycoproteins is their expression in *Komagataella phaffii*, a yeast strain that attaches high-mannose-type glycans [8, 9] and was previously termed *Pichia pastoris*, until sequence analysis of rRNA subunits revealed *K. phaffii* as correct designation for this strain [10, 11]. Because of its potential to target C-type lectin receptors, fungal glycosylation is considered to be immunogenic [12, 9, 13], although direct evidence for this hypothesis remains elusive. Moreover, in vaccination studies, *K. phaffii*-derived antigens are used in combination with adjuvants [14–19], but none of these studies covered a direct comparison between N-glycosylated vs. non-glycosylated proteins *in vivo*. Additionally, these studies mainly focused on proteins that are already glycosylated in their natural state like Hemagglutinin [15, 17] and Ovalbumin (OVA) [14, 20], or did not further investigate a specific role of glycosylation [16, 18, 19].

In this study, we directly monitored the effect of N-glycosylation on antigen immunogenicity compared to the de-glycosylated protein *in vivo* and assessed the immunogenic properties of N-glycosylated proteins *per se*. Therefore, we targeted non-glycosylated proteins towards the MR by introduction of a single N-glycosylation site and expression in *K. phaffii*. Whereas N-glycosylation did not influence DC maturation, a beneficial effect of N-glycosylation on antigen-specific T cell responses *in vivo* depended on the nature of the antigen.

## RESULTS

### Glycan-dependent binding of the MR to non-glycoproteins after expression by K. phaffii

**Figure 1:**
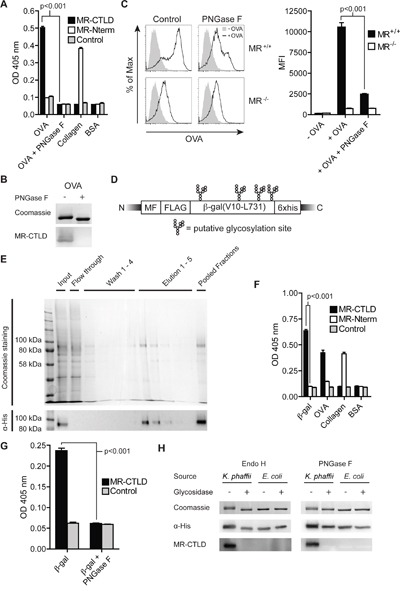
Glycan-dependent binding of the MR to β-gal expressed in K. phaffii **A**. Binding of MR-CTLD, MR-Nterm or the Fc part of a human IgG isotype control to OVA, PNGase-treated OVA, collagen or BSA was determined by ELISA. **B**. OVA and PNGase F-treated OVA were separated by SDS-PAGE and subjected to coomassie staining or far-western blot analysis using 50 μg/ml MR-CTLD. **C**. BM-DCs were incubated with fluorochrome-labeled OVA or PNGase F-treated OVA and analyzed by flow cytometry. **D**. Scheme of β-gal expressed by *K. phaffii*. MF: Mating Factor alpha leader sequence, FLAG: FLAG-epitope. **E**. Purification of β-gal(V10-L731) from *K. phaffii* by affinity chromatography. **F**. Binding of MR-CTLD, MR-Nterm or isotype control to β-gal, OVA, collagen or BSA was determined by ELISA. **G**. as in F) using glycosylated or de-glycosylated β-gal. **H**. Far-western blot analysis using MR-CTLD on untreated, PNGase F-treated Endo H-treated β-gal from *K. phaffii* and *E. coli*. Bar graphs show mean values ± SEM. All graphs depict representative examples of at least 3 independent experiments.

Since the model antigen OVA is internalized very efficiently by the MR [21], we first analyzed whether binding of OVA to the MR was mediated by glycosylation. Therefore, we performed a binding assay using chimeric proteins consisting of the Fc region of a human IgG1 attached to the CTLD4-7 of the MR (MR-CTLD) or to the N-terminal region of the MR consisting of the CR, FN II region and CTLD1-2 (MR-Nterm) [22]. We could demonstrate that recognition of OVA is specifically mediated by MR-CTLD (Figure 1A), whereas MR-Nterm recognized collagen as previously described [22]. Such binding was confirmed by far western blot using MR-CTLD (Figure 1B). Since CTLD4-7 are responsible for recognition of mannosylated glycans [4, 23], we treated OVA with the glycosidase PNGase F and demonstrated that recognition of OVA by MR-CTLD indeed was mediated by glycans (Figure 1A and 1B). Additionally, MR-mediated uptake of OVA by bone marrow-derived DCs (BM-DCs) was inhibited when OVA was de-glycosylated, confirming glycan-dependent binding of the MR to OVA (Figure 1C).

Next, we intended to investigate whether it is possible to target proteins that are non-glycosylated in their native state toward the MR by expression in *Komagataella phaffii*, a yeast strain generating high-mannose-type glycans [8, 9]. Therefore, we first expressed a secreted form of β-galactosidase (amino acids V10-L731), a non-glycosylated bacterial protein with 4 putative N-glycosylation sites (Figure 1D), in *K. phaffii* and purified the protein from the supernatant by affinity chromatography (Figure 1E). Binding assays demonstrated that purified β-gal indeed was recognized by MR-CTLD (Figure 1F). Such binding was prevented in the presence of mannan, a competitive inhibitor of MR-mediated endocytosis, or in the absence of calcium (Supplementary Figure 1), pointing out that binding to β-gal indeed depended on the C-type lectin activity of the MR. Consistently, MR binding was abrogated after treatment of β-gal with PNGase F or Endo H (Figure 1G, 1H). Furthermore, β-gal expressed in and purified from *E. coli* was not recognized by MR-CTLD (Figure 1H). These data demonstrate that *K. phaffii* indeed glycosylates putative N-glycosylation sites in proteins that are normally non-glycosylated and thereby creates ligands of the MR.

### MR-mediated uptake of mannosylated β-gal and targeting in early endosomes

**Figure 2:**
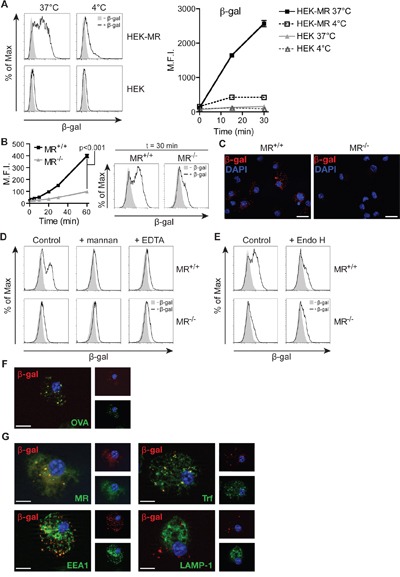
MR-mediated uptake of β-gal expressed in K. phaffii **A**. Internalization of β-gal from *K. phaffii* by HEK-MR or control HEK293T (HEK) cells monitored by flow cytometry. **B** and **C**. Internalization of β-gal by wildtype or MR-deficient BM-DCs monitored by flow cytometry (B) or fluorescence microscopy (C). Nuclei (blue) were visualized with 1 μg/ml 4',6-diamidino-2-phenylindole (DAPI). Bars: 20 μm. **D**. Uptake of β-gal by wildtype BM-DCs in the presence of 3 mg/ml mannan or 5 mM EDTA. **E**. Uptake of untreated or Endo H-treated β-gal by wildtype or MR-deficient BM-DCs. **F**. BM-DCs were incubated with fluorochrome-labeled β-gal and OVA and analyzed by fluorescence microscopy. Nuclei are depicted in blue. Bars: 10 μm. **G**. Co-localization of β-gal with Trf, EEA1, the MR and LAMP-1 depicted by fluorescence microscopy. Graphs show mean values ± SEM. All graphs depict representative examples of at least 3 independent experiments.

Subsequently, we monitored whether recognition of glycosylated β-gal by the MR resulted in enhanced receptor-mediated endocytosis. Therefore, we incubated a MR-expressing HEK293T cell line (HEK-MR) [24] or control HEK293T cells (HEK) with fluorochrome-labeled β-gal. Increased uptake of β-gal was only observed in cells expressing the MR (Figure 2A). Next, we analyzed endocytosis of glycosylated β-gal BM-DCs, which are well equipped to internalize and present extracellular antigens. Efficient uptake of β-gal was observed in wildtype but not in MR-deficient DCs (Figure 2B, 2C) and was prevented in the presence of mannan or the calcium chelator EDTA (Figure 2D). Importantly, de-glycosylation of β-gal reduced uptake in wildtype DCs to a level similar to MR-deficient DCs (Figure 2E), demonstrating that the MR indeed internalized β-gal after recognition of its glycans.

In order to be cross-presented efficiently, MR-internalized OVA needs to be targeted towards an early endosomal compartment, from where processing takes place [1, 25]. Therefore, we analyzed whether β-gal is targeted towards the same compartment after MR-mediated endocytosis. Fluorescence microscopy of BM-DCs after co-incubation with OVA and β-gal demonstrated that both antigens indeed are localized in the same intracellular compartments (Figure 2F). Additionally, β-gal co-localized with the MR, Transferrin (Trf) and the early endosome antigen 1 (EEA1) (all markers for OVA^+^ early endosomes [1]) but not with the lysosome-associated membrane protein 1 (LAMP-1) (Figure 2G).

These findings show that glycosylated β-gal secreted by *K. phaffii* is a specific ligand of the MR and is targeted towards an early endosomal compartment related to cross-presentation.

### Targeting of proteins lacking putative N-glycosylation sites toward the MR

**Figure 3:**
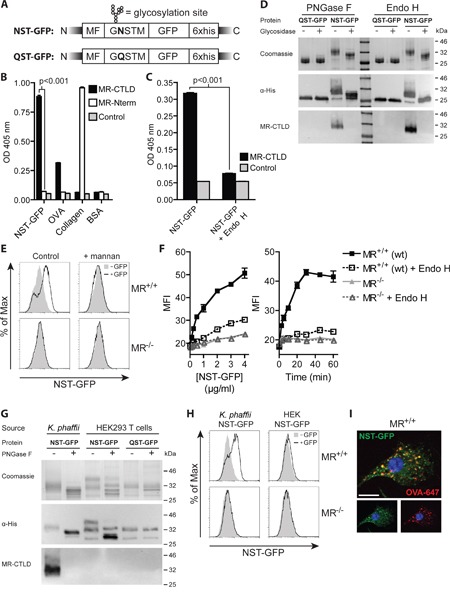
Introduction of an artificial N-glycosylation site in GFP and expression in K. phaffii results in binding and endocytosis by the MR **A**. Scheme of *K. phaffii* -expressed GFP constructs. MF: Mating Factor alpha leader sequence. **B**. Binding of MR-CTLD, MR-Nterm or isotype control to NST-GFP, OVA, collagen or BSA depicted by ELISA. **C**. Binding of MR-CTLD to untreated or Endo H-treated NST-GFP by ELISA. **D**. Binding of MR-CTLD to untreated, PNGase F-treated or Endo H-treated NST-GFP or QST-GFP depicted by far-western blot analysis. **E**. Uptake of NST-GFP by wildtype or MR-deficient DCs in the presence or absence of 3 mg/ml mannan. **F**. Uptake of untreated or Endo H-treated NST-GFP by wildtype or MR-deficient BM-DCs. **G**. Binding of MR-CTLD to untreated or PNGase F-treated NST-GFP or QST-GFP from *K. phaffii* or HEK293T cells depicted by far-western blot analysis. **H**. Uptake of NST-GFP from *K. phaffii* and HEK293T cells by wildtype and MR-deficient DCs. **I**. Fluorescence microscopy of BM-DCs incubated with NST-GFP and fluorochrome-labeled OVA. Nuclei are depicted in blue. Bar: 10 μm. Graphs show mean values ± SEM. All graphs depict representative examples of at least 3 independent experiments.

Since not all proteins bear putative N-glycosylation sites, we investigated in a next step whether it is possible to target proteins lacking such sites toward the MR by introduction of a single N-X-S/T glycosylation motif. To answer this question, we chose GFP, a protein which lacks putative N-glycosylation sites and N-terminally introduced the amino acids GNSTM (NST-GFP, Figure 3A), which we optimized for efficient N-glycosylation [26–29]. After purification of this protein from *K. phaffii*, we observed a specific binding to MR-CTLD (Figure 3B), which again was prevented in the presence of mannan or in the absence of calcium (Supplementary Figure 2). Importantly, binding of the MR was prevented after de-glycosylation or after replacement of asparagine by glutamine in the glycosylation site (QST-GFP) (Figure 3C, 3D). Consistently, glycosylated GFP was efficiently internalized by wildtype but not by MR-deficient DCs (Figure 3E). Such internalization was strongly reduced in the presence of mannan (Figure 3E) or by treatment with Endo H (Figure 3F). Glycosylation of GFP did neither change its excitation and emission maximum nor increased its emission at 540 nm (Supplementary Figure 3), excluding that alterations in GFP signal after uptake were due to changes in its fluorochrome properties. Furthermore, GNSTM-tagged GFP expressed in and purified from HEK293T cells was neither recognized by MR-CTLD (Figure 3G) nor internalized by MR-expressing DCs (Figure 3H), pointing out that indeed the high-mannose glycans attached by *K. phaffii* were crucial for MR recognition. Finally, we demonstrated that NST-GFP internalized by BM-DCs co-localized with OVA (Figure 3I), pointing out that also GFP is targeted towards early endosomes related to cross-presentation.

Taken together, these findings prove that the introduction of a single N-glycosylation site and expression in *K. phaffii* leads to efficient N-glycosylation, resulting in specific recognition and internalization by the MR.

### Treatment with N-glycosylated proteins does not lead to DC maturation

**Figure 4:**
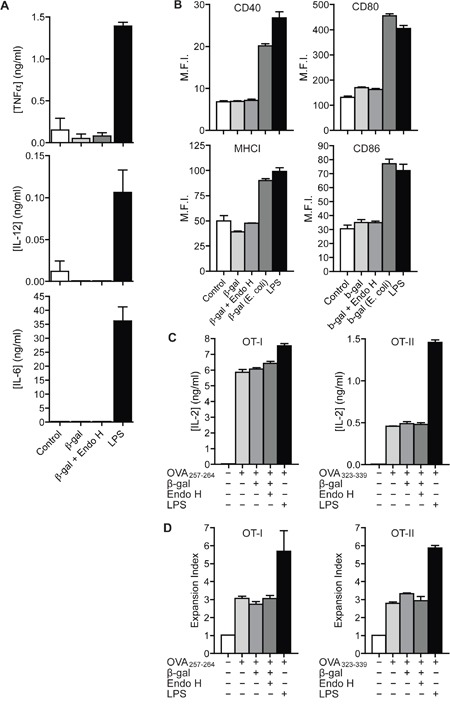
Recognition of N-glycosylated proteins does not influence DC maturation **A**. BM-DCs were treated with 100 μg/ml β-gal, 100 μg/ml Endo H-treated β-gal or 0.1 μg/ml LPS. Secretion of TNFα, IL-12 and IL-6 was determined after 18h by ELISA. **B**. BM-DCs were treated with 4 μg/ml untreated β-gal or Endo H-treated β-gal from *K. phaffii*, with 4 μg/ml β-gal from *E. coli* or with 1 μg/ml LPS. Expression of MHC I, CD40, CD80 and CD86 was monitored after 18 h by flow cytometry. **C**. BM-DCs were stimulated with untreated β-gal, Endo H-treated β-gal, LPS or medium only. After intense washing, cells were pulsed with OVA_257-264_ (left) or OVA_323-339_ (right) and incubated with OT I (left) or OT II (right) T cells. T cell activation was measured after 18 h by ELISA. **D**. Identical to C) using CFSE-labeled T cells. T cell proliferation was measured after 3 days by flow cytometry. Graphs show mean values ± SEM of three technical replicates. Graphs depict representative examples of at least 3 independent experiments.

To investigate whether treatment with *K. phaffii-* derived N-glycosylated antigens *per se* leads to DC activation, we first treated DCs with N-glycosylated or de-glycosylated β-gal and analyzed the secretion of inflammatory cytokines. Whereas addition of LPS resulted in clear induction of TNFα, IL-12 and IL-6, none of these cytokines was induced after treatment with β-gal, irrespective of its glycosylation status (Figure 4A). Also the expression of MHC I molecules and of the co-stimulatory molecules CD40, CD80 and CD86 remained unaffected by addition of β-gal (Figure 4B). To fully exclude an effect of such glycosylated proteins on DC maturation and their general capacity to activate T cells, we pre-treated BM-DCs with glycosylated or de-glycosylated β-gal and subsequently pulsed them with OVA_257-264_ or OVA_323-339_ before adding OT I or OT II T cells, respectively. Addition of β-gal influenced neither IL-2 secretion (Figure 4C) nor T cell proliferation (Figure 4D), regardless of its glycosylation state, whereas both IL-2 secretion and T cell proliferation were clearly increased after addition of LPS. Additionally, we didn't observe any differences in DC migration towards a CCL19 gradient after their treatment with glycosylated or de-glycosylated proteins (Supplementary Figure 4). These data demonstrate that recognition of N-glycosylated proteins derived from *K. phaffii* do not influence the activation state of DCs *per se*.

### T cell activation by N-glycosylated proteins *in vivo*

**Figure 5:**
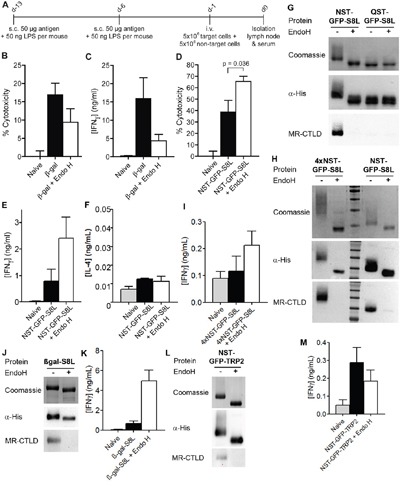
Effect of N-glycosylation on antigen presentation critically depends on the nature of the antigen **A**. Scheme of *in vivo* experiments. s.c.: subcutaneously, i.v.: intravenously. **B**. Mice were immunized with N-glycosylated or de-glycosylated β-gal as depicted in A). β-gal-specific cytotoxicity was determined after injection of a mixture of differentially labeled target and control cells by flow cytometry (n=28). **C**. Cells from draining lymph nodes from mice treated as in A) were isolated and re-stimulated with 2 μM of the β-gal epitope ICPMYARV for 18 h. Secretion of IFNγ in the supernatant was determined by ELISA (n=12). **D**. Mice were immunized with N-glycosylated or de-glycosylated NST-GFP-S8L as in A). Cytotoxicity was determined by flow cytometry (n=6). **E** and **F**. Mice were treated as in D) and IFNγ (E) or IL-4 (F) secretion was determined after re-stimulation with SIINFEKL (n=6). **G**. Binding of MR-CTLD to untreated or Endo H-treated NST-GFP-S8L and QST-GFP-S8L (purified from *K. phaffii*) depicted by far-western blot analysis. **H**. Binding of MR-CTLD to untreated or Endo H-treated 4xNST-GFP-S8L and NST-GFP-S8L depicted by far-western blot analysis. **I**. Mice were immunized with untreated or Endo H-treated 4xNST-GFP-S8L as in A). Secretion of IFNγ after re-stimulation with SIINFEKL was determined by ELISA (n=6 per group). **J**. As in H using β-gal-S8L. **K**. As in I) but with β-gal-S8L (n=6). **L**. As in H using NST-GFP-TRP2. **M**. As in I but after injection of NST-GFP-TRP2 and restimulation with SVYDFFVWL (n=6).

Next, we intended to investigate whether N-glycosylation introduced by expression in *K. phaffii* resulted in enhanced antigen presentation *in vivo*. We therefore purified glycosylated β-gal as described above (Figure 1D, 1E) and immunized recipient mice by s.c. injection of either glycosylated or Endo H-treated β-gal as indicated in Figure 5A. To monitor β-gal-specific activation of endogenous β-gal specific CD8^+^ T cells, we injected a mixture of differentially labeled control cells or β-gal-specific target cells that were loaded with the peptide ICPMYARV, which has previously been shown to be a potent H2-Kb-restricted internal epitope of β-gal [30]. Flow cytometric analysis revealed a clear specific elimination of the target cells in mice immunized with glycosylated β-gal, which was markedly though not significantly reduced in mice that received de-glycosylated β-gal (Figure 5B). Similarly, we observed an increase in IFNγ secretion when re-stimulating cells from the draining lymph node only after immunization with glycosylated β-gal (Figure 5C). To analyze whether increased cross-presentation *in vivo* is a general feature of N-glycosylated antigens, we additionally investigated activation of endogenous CD8^+^ T cells that were generated *in vivo* against NST-GFP fused to the OVA-epitope SIINFEKL (NST-GFP-S8L). Surprisingly, we didn't observe any beneficial effect of N-glycosylation compared to the de-glycosylated protein on the cytotoxic activity (Figure 5D) or on the secretion of IFNγ (Figure 5E) by endogenous CD8^+^ T cells. In contrast, T cell activation and secretion of IFNγ were clearly suppressed compared to T cells from animals that were immunized with the de-glycosylated antigen, whereas the secretion of IL-4 remained unaltered (Figure 5F). These data demonstrate that increased cross-presentation by N-glycosylation *in vivo* is not a general feature but seems to be restricted to specific antigens.

Next, we investigated whether impaired T cell activation after injection of glycosylated NST-GFP-S8L might have been due to alterations (N-glycosylation) within the SIINFEKL epitope. Therefore, we purified NST-GFP-S8L and QST-GFP-S8L from *K. phaffii* and either treated these proteins with Endo H or not. Subsequent western blot analysis of these proteins revealed that only NST-GFP-S8L was glycosylated and recognized by the MR and that this recognition by was abrogated after Endo H treatment (Figure 5G). Importantly, QST-GFP-S8L was not bound by the MR, regardless whether it was treated with Endo H or not (Figure 5G). If however, the SIINFEKL epitope would be N-glycosylated, it should have been glycosylated in QST-GFP-S8L too and QST-GFP-S8L should have been recognized by the MR. Therefore, it can be concluded that the SIINFEKL epitope within NST-GFP-S8L was not N-glycosylated itself after purification from *K. Phaffii*.

Since β-gal, as described above, contains four putative internal N-glycosylation sites and NST-GFP-S8L contains only one N-terminal N-glycosylation site, we next investigated whether the observed differences in T cell activation between β-gal and NST-GFP-S8L were due to a different degree of antigen N-glycosylation. To this end, we generated a GFP-S8L protein containing four N-glycosylation sites, of which two were positioned at its N-terminus and two at its C-terminus (4xNST-GFP-S8L), and purified this protein from *K. phaffii*. Introduction of four N-glycosylation sites resulted in a clearly enhanced degree of glycosylation compared to NST-GFP-S8L (Figure 5H). Similar to our results with NST-GFP-S8L, immunization of recipient mice with 4xNST-GFP-S8L resulted in a superior antigen-specific CD8^+^ T cell activation after immunization with the de-glycosylated antigen compared to the glycosylated protein (Figure 5I). These results clearly demonstrate that the observed differences in T cell activation after immunization with (de-)glycosylated β-gal or GFP were not due to a differential degree of antigen N-glycosylation.

Subsequently, we analyzed whether the investigated epitope rather than the glycosylated antigen determined the level of T cell activation. To investigate this question, we generated a fusion protein between β-gal and the SIINFEKL epitope (β-gal-S8L) and purified it from *K. phaffii* (Figure 5J). Whereas treatment with glycosylated β-gal resulted in a clearly enhanced β-gal-specific CD8^+^ T cell response (Figure 5B, 5C), the activation of endogenous SIINFEKL-specific T cells after immunization with glycosylated β-gal-S8L was clearly impaired compared to the de-glycosylated protein (Figure 5K), suggesting that not the glycosylated carrier protein but rather the investigated epitope (in this case SIINFEKL) determined the functionality of the activated cytotoxic T cells. To finally test this hypothesis, we generated a fusion protein between NST-GFP and the H2-Kb epitope SVYDFFVWL from the murine tyrosinase-related protein 2 (TRP2) [31] (NST-GFP-TRP2, Figure 5L). In contrast to our observations with the SIINFEKL epitope from NST-GFP-S8L, T cell activation by DCs treated with NST-GFP-TRP2 was superior compared to its de-glycosylated form (Figure 5M), proving that the properties of the epitope decisively contributed to the effect of glycosylation on CD8^+^ T cell activation.

**Figure 6:**
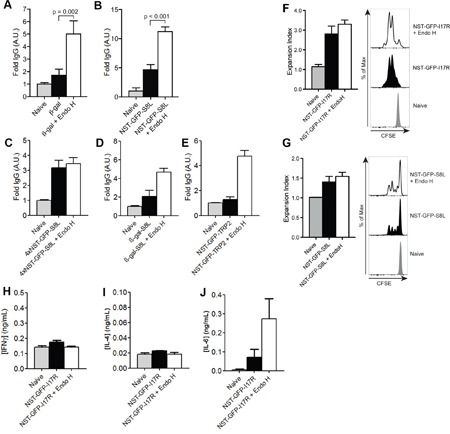
Influence of N-glycosylation on the induction of a humoral immune response **A-E**. IgG against β-gal (A), NST-GFP-S8L (B), 4xNST-GFP-S8L (C), β-gal-S8L (D) or NST-GFP-TRP2 (E) from the serum of mice treated as in Figure 5A was determined by ELISA (n≥6). **F** and **G**. Mice were injected with CFSE-labeled OT II (F) or OT-I (G) and challenged with NST-GFP-I17R (F) or NST-GFP-S8L (G). T cell proliferation was monitored by flow cytometry after 3 days. **H-J**. Mice were immunized with untreated or Endo H-treated NST-GFP-I17R as indicated in Figure 5A. Secretion of IFNγ (H), IL-4 (I) or IL-6 (J) secretion was determined by ELISA after re-stimulation with ISQAVHAAHAEINEAGR (n=6). All graphs show mean values ± SEM.

Additionally, we analyzed the influence of N-glycosylation on the induction of a humoral immune response. Therefore, we monitored the concentrations of β-gal-specific IgG in the blood of mice treated as depicted in Figure 5A. We monitored a strong IgG response after injection of de-glycosylated β-gal (Figure 6A). Surprisingly, the induction of β-gal-specific IgG after immunization with N-glycosylated β-gal was hardly detectable (Figure 6A), pointing out that N-glycosylation prevents MHC II-restricted presentation of β-gal. Similar observations were made after immunization with N-glycosylated or de-glycosylated NST-GFP-S8L (Figure 6B), 4xNST-GFP-S8L (Figure 6C), β-gal-S8L (Figure 6D) or NST-GFP-TRP2 (Figure 6E), demonstrating that for all antigens and epitopes tested in this study, the generation of a humoral immune response was decreased by N-glycosylation.

Subsequently, we analyzed whether such differential antibody production might be due to differences in CD4^+^ T cell activation. To investigate this question, we generated an NST-GFP construct containing the MHC II-restricted OVA epitope ISQAVHAAHAEINEAGR (OVA_323-339_) (NST-GFP-I17R). First, we challenged mice with untreated or Endo H-treated NST-GFP-I17R and monitored *in vivo* T cell proliferation after injection of CFSE-labeled OVA-specific CD4^+^T (OT II) cells. Only marginal differences in CD4^+^ T cell proliferation could be observed after injection of glycosylated or de-glycosylated NST-GFP-I17R (Figure 6F). Remarkably, despite clear differences in cytotoxic activity and secretion of IFNγ after injection of untreated or de-glycosylated NST-GFP-S8L (Figure 5D, 5E), the effect on CD8^+^ T cell proliferation was similar (Figure 6G).

Finally, we investigated the secretion of cytokines by CD4^+^ T cells after immunization of NST-GFP-I17R and re-stimulation with the corresponding MHC II peptide (OVA_323-339_). Whereas no differences in secretion of IFNγ (Figure 6H) or IL-4 (Figure 6I) could be detected, a clear induction of IL-6 was observed after immunization of de-glycosylated NST-GFP-I17R (Figure 6J). Since this cytokine was originally identified as B cell growth factor and plays an important role in antibody production and class switching *in vivo* [32, 33], the results point out that impaired generation of an antibody response after injection of extensively N-glycosylated antigens might be due to diminished secretion of IL-6 by CD4^+^ T cells.

## DISCUSSION

In this study, we demonstrated that non-glycosylated proteins can be targeted towards the MR either by the usage of intrinsic N-X-S/T motifs or by the introduction of an artificial N-glycosylation site and subsequent expression in *K. phaffii*. Introduction of a single N-glycan was sufficient for efficient binding to the CTLD region of the MR, which generally is responsible for binding mannosylated glycoproteins, including the model antigen OVA. *K. phaffii* does not only generate high-mannose glycans, but also allows a straightforward purification of secreted proteins from the culture medium and avoids the necessity to elaborately conjugate purified antigens to carrier proteins (like antibodies) [14, 5] for targeting C-type lectin receptors.

In several studies, fungal mannosylation is considered to be immunogenic because of its potential to target C-type lectin receptors [12, 9, 13]. However, in most vaccination studies, *K. phaffii*-derived antigens are used in combination with adjuvants [14, 15, 17, 18], although one study reports an immunostimulatory effect of *K. phaffii-* derived proteins also in the absence of adjuvant [19]. Interestingly, we didn't observe any immunostimulatory effect of *K. phaffii-*derived antigens on BM-DCs. We could neither detect differences in expression of co-stimulatory molecules nor alterations in T cell functionality after priming by DCs treated with proteins from *K. phaffii* or differences in DC migration. These observations are in line with a study of Luong and colleges, demonstrating the absence of an immunostimulatory response after DC treatment with *K. phaffii*-derived OVA [34]. The reason for the missing inflammatory response of DCs remains speculative. As demonstrated in this study, N-glycosylated antigens target the MR, which lacks signaling motifs in its cytoplasmic tail [35]. However, it is thinkable that the immunostimulatory properties of fungal proteins reported before are due to O-glycosylation, as a high degree of O-mannosylation leads to recognition by e.g. DC-SIGN, which can induce DC maturation due to its intracellular signaling motif [36].

Since a direct comparison of the immunogenic properties of N-glycosylated versus non-glycosylated antigens *in vivo* was missing, the influence of N-glycosylation on antigen presentation *in vivo* remained elusive. Here, we investigated this open question by purifying N-glycosylated antigens from *K. phaffii* followed by de-glycosylation. By this approach, we could circumvent differences in DC activation due to expression of non-glycosylated proteins in e.g. *E. coli* as described previously [37]. Since proteins purified from bacteria strongly activate DCs (Figure 4B), it is impossible in such approaches to distinguish whether putative alterations in T cell activation are due to differences in antigen glycosylation or rather to altered stimulatory properties by contaminations like LPS.

Immunization of mice with N-glycosylated β-gal resulted in increased CD8^+^ T cell functionality *in vivo* as monitored by increased cytotoxicity and enhanced secretion of IFNγ. However, a beneficial effect of N-glycosylation on cross-presentation was not observed for NST-GFP-S8L. In fact, activation of CD8^+^ T cells after injection of Endo H-treated NST-GFP-S8L was even increased compared to N-glycosylated antigen. We could show that these differences were neither due to internal N-glycosylation of the SIINFEKL epitope nor to a differential degree of N-glycosylation of these proteins. Surprisingly, we demonstrated that the observed differences in CD8^+^ T cell activation were closely related to the analyzed epitope and that the induction of especially a SIINFEKL-specific CD8^+^ T cell response was impaired by antigen N-glycosylation. The reasons for these observations remain elusive. However, since we monitored the induction of endogenous antigen-specific T cells in our functional assays, it cannot be explained by altered characteristics of adoptively transferred T cells. Additionally, as mentioned above, we could exclude internal N-glycosylation of the SIINFEKL epitope as a reason for impaired T cell functionality. Also modifications other than N-glycosylation (e.g. O-glycosylation,…) are very unlikely to have caused the restricted presentation of the SIINFEKL epitope. If such modifications should have masked the epitope, thereby preventing its loading onto MHC I molecules or its recognition by the T cell receptor, one would expect them to do this in both untreated und Endo H-treated proteins, since Endo H is not able to remove such modifications. In principle, it is thinkable that the SIINFEKL epitope, which in all our constructs was present at the very C-terminus, is either directly recognized by other receptors or induces conformational changes that lead to recognition by other receptors. Such an effect would target the antigen away from the MR and could possibly lead to MR- and hence N-glycosylation-independent cross-presentation. Additionally, whereas MR-mediated endocytosis of glycosylated antigens targets them towards a non-degradative early-endosomal compartment, non-glycosylated proteins are targeted towards vesicles containing activated cathepsins. These proteases could possible cleave off and release the C-terminal SIINFEKL epitope. Since SIINFEKL is a very strong epitope with very high affinity for H2-Kb, such release could lead to direct loading on MHC I molecules within these compartments. This process is also known as the vacuolar (TAP-independent) cross-presentation pathway [38, 39]. However, whether such mechanisms indeed are responsible for the differential effect of the SIINFEKL epitope on CD8^+^ T cell activation remains speculative and needs to be investigated in future experiments.

Additionally, we demonstrated that the generation of a strong humoral immune response was only observed after immunization with de-glycosylated antigens. It is possible that N-glycosylation of putative epitopes prevents binding of cleaved peptides onto MHC II molecules or recognition by the T cell receptor as demonstrated before [40], both resulting in impaired CD4^+^ T cell activation. Beyond that, decreased MHC II-restricted presentation after injection of N-glycosylated compared to de-glycosylated β-gal might be partially explained by the fact that proteins internalized by the MR are processed predominantly for cross-presentation and that the MR is dispensable for efficient MHC II-restricted presentation [1, 2]. Accordingly, internalization for presentation on MHC II has been shown to be mediated by fluid phase pinocytosis or additional endocytic receptors distinct from the MR [1]. Finally, we could demonstrate that immunization with N-glycosylated proteins inhibits the secretion of IL-6 by CD4^+^ T cells. Previous studies already revealed that cross-linking of the MR on DCs alters their stimulatory capacities. In particular, it was shown that MR cross-linking on DCs directly influenced the functionality of activated CD4^+^ T cells, altering their cytokine profile into a rather immune-suppressive state without critically influencing their proliferation [41]. Therefore, it is thinkable that the N-glycosylated antigens used in this study caused a similar cross-linking of the MR and that the concomitant CD4^+^ T cell were reprogrammed into a regulatory phenotype, resulting in the impaired secretion of IL-6. Since IL-6 has been demonstrated before to play a crucial role in antibody production and class switching *in vivo* [32, 33], MR cross-linking by N-glycosylated antigens might directly be responsible for impaired antibody responses observed in this study.

Taken together, we demonstrated that the introduction of artificial N-glycosylation by *K. phaffii* is sufficient to target proteins that are non-glycosylated under normal conditions towards the MR. We showed that introduction of a single N-X-S/T motif in proteins that lack putative N-glycosylation sites was sufficient for efficient binding to the MR. However, N-glycosylated proteins did not have an immunostimulatory effect on the DC *per se* and a beneficial effect of N-glycosylation on antigen presentation *in vivo* was critically dependent on the nature of the epitope. Therefore, for vaccination studies, the effect of glycosylation on T cell activation must be carefully evaluated for individual antigens.

## MATERIALS AND METHODS

### Antibodies, reagents and mice

Armenian Hamster anti-CD80 (16-10A1), mouse anti-MHC I (28-14-8) and rat anti-CD40 (1C10) were from eBioscience (Frankfurt, Germany), rat anti-MR (MR5D3), mouse anti-His (AD1.1.10) and rat anti-CD86 (PO3) from AbD Serotec (Puchheim, Germany), rat anti-LAMP-1 (1D4B) from BD, rabbit anti-EEA1 (H-300) and all secondary antibodies from Santa Cruz (Heidelberg, Germany).

Fluorochrome-labeled OVA was from Thermo Scientific (Darmstadt, Germany), unlabeled OVA from Sigma (Taufkirchen, Germany), OVA_257-264_ and OVA_323-339_ from Tebu-bio (Offenbach, Germany), β-gal_497-504_ (ICPMYARV) from PSL Peptide (Heidelberg, Germany). PNGase F and Endo H were obtained from New England Biolabs (Frankfurt, Germany).

Wildtype C57BL/6J mice, MR-deficient mice, OT I and OT II mice were bred under specific pathogen-free conditions and used in accordance with local animal experimentation guidelines. Mouse studies were approved by local regulatory agencies (Landesamt fuer Natur, Umwelt und Verbraucherschutz Nordrhein-Westfalen - LANUV NRW; Approval number §84-02.04.2012.A264). For all experiments, mice between 8 and 16 weeks of age were used. All mice were anaesthetized before euthanization by cervical dislocation.

### Expression and purification of β-gal and GFP from *K. phaffii*

β-gal(V10-L731) and GFP were cloned into the pPICZα expression vector. Introduction of an artificial N-glycosylation site into pPICZ α was performed using CAGCATCGATGGGTAATTCTACTATGGGTACCTGCAGTCGACTTCTAGACTCGAGTAGC and GCTACTCGAGTCTAGAAGTCGACTGCAGGTACCCATAGTAGAATTACCCATCGATGCTG (NST) or CAGCATCGATGGGTCAATCTACTATGGGTACCTGCAGTCGACTTCTAGACTCGAGTAGC and GCTACTCGAGTCTAGAAGTCGACTGCAGGTACCCATAGTAGATTGACCCATCGATGCTG (QST) as oligos. The OVA epitope SIINFEKL was introduced into NST-GFP-S8L using TATGTCAGGCCTTGAGCAGCTTGAGAGTATAATCAACTTTGAAAAACTGACTGAATGGACCAGTTCTT and CTAGAAGAACTGGTCCATTCAGTCAGTTTTTCAAAGTTGATTATACTCTCAAGCTGCTCAAGGCCTGACA. 4xNST-GFP was constructed using CTGAGCATCGATGGGTAATGGTACTGGTCATGGCAATTCTACTATGGGTACCTAGC, GCTAGGTACCCATAGTAGAATTGCCATGACCAGTACCATTACCCATCGATGCTCAG, GCTACATATGGGTAATGGTACTGGTCATGGCAATTCTACTATGGGATCTAGACTAG and GCTACATATGGGTAATGGTACTGGTCATGGCAATTCTACTATGGGATCTAGACTAG as primers, NST-GFP-TRP2 using GTTCCAATTGACAAGCTTTTG and GCGCGGTACCGAGCCACACAAAAAAGTCATACACGCTTCCTGCTCCTCCTGCTCCCATAGTAGAATTACCCATCG as primers. Afterwards, expression plasmids were introduced in *K. phaffii* strain KM71H. Biomass production occurred in BMGY. Expression of recombinant proteins was induced in 10% of the starting volume using 2% methanol in sodium phosphate buffer (300 mM NaPO_4_, 150 mM NaCl2, pH8). After 72h, recombinant proteins from the supernatant were purified using Ni-NTA affinity chromatography. Subsequently, proteins were concentrated and buffer was exchanged to PBS using centrifugal filter units (Merck Millipore) with a cutoff of 30 kDa (β-gal) or 10 kDa (GFP). For purification of NST-GFP, NST-GFP-S8L and QST-GFP, proteins were additionally purified by size exclusion chromatography (Sephacryl S200 HR, Sigma, Taufkirchen, Germany).

### Expression and purification of NST-GFP and QST-GFP from HEK293T cells

NST-GFP and QST-GFP were cloned behind the IL-2 secretion sequence of pIgFuse, thereby removing the IgG sequence, and transfected into HEK293T cells. After 24 h, medium was exchanged. After another 72 h, supernatant was collected and proteins were purified as described above.

### Purification of β-gal from *E. coli*

β-gal(V10-L731) was cloned into the bacterial expression vector pQE60. Expression in the *E.coli* strain XL1-blue was induced using 0.1 mg/ml Isopropyl-β-D-thiogalactopyranosid (IPTG) for 18h. The bacterial pellet was lysed by sonication and recombinant proteins were purified as described above.

### De-glycosylation using PNGase F or Endo H

De-glycosylation was performed according to the manufacturers’ guidelines. Briefly, for de-glycosylation with PNGase F, glycosylated proteins were denatured and incubated with 100 Units of PNGase F / mg protein for 18 h at 37°C. For de-glycosylation with Endo H, native proteins were incubated with 250 Units of Endo H / mg protein for 18 h at 37°C. For all assays, control samples were treated identically but without addition of enzyme.

### Generation of BM-DCs

BM-DCs were generated using the supernatant of a GM-CSF-producing cell line as described before [1].

### Uptake of fluorochrome-labeled antigens

β-gal from *K. phaffii* was fluorochrome-labeled using the Alexa Fluor 647 Protein Labeling Kit (Thermofischer, Darmstadt, Germany). BM-DCs or HEK cells were incubated with 250 ng/ml fluorochrome-labeled OVA (Thermofischer, Darmstadt, Germany), 500 ng/ml fluorochrome-labeled β-gal or 500 ng/ml (unlabeled) GFP for 20 min. Cells were harvested and antigen uptake monitored by flow cytometry.

### Generation and purification of MR-CTLD and MR-Nterm

MR-CTLD (encompassing CTLD4-7 fused to the Fc region of hIgG1) or MR-Nterm (encompassing the CR region, the FN II domain and CTLD1-2 fused to the Fc region of hIgG1) proteins were generated as described before [22]. As isotype control, the Fc region of hIgG1 was used.

### Binding of MR subdomains by ELISA

0,1 mg/ml antigen was coated on an ELISA plate for 1 h. After washing and blocking with 0,3 % BSA, 10 μg/ml of recombinant MR-CTLD, MR-Nterm or isotype control (Fc from human IgG1) were added. After incubation with an alkaline phosphatase-conjugated anti-human IgG secondary antibody and addition of 1 mg/ml p-Nitrophenylphosphate, binding was monitored by colorimetric analysis at 405 nm.

### Far-western blot analysis using MR-CTLD

Antigens were loaded on an SDS-gel (1 μg/ lane), blotted on a nitrocellulose membrane and incubated with 50 μg/ml MR-CTLD for 18 h in TBST containing 10 mM CaCl_2_. Far-western blot analysis was performed after addition of a horseradish peroxidase-conjugated secondary antibody.

### Immunofluorescence microscopy

BM-DCs were incubated with 500 ng/ml fluorochrome-labeled OVA, β-gal or Trf for 20 min and chased with medium for another 20 min. Afterwards, cells were fixed in 4 % paraformaldehyde and stained with antibodies against EEA1, the MR and LAMP-1 and secondary antibodies as described before [42]. Nuclei were visualized with 1 μg/ml 4',6-diamidino-2-phenylindole (DAPI). Samples were analyzed with an ApoTome microscope (Zeiss, Oberkochen, Germany).

### Transwell migration assay

At day 7 of culture, BM-DCs were stimulated to mature by adding 200 ng/ml LPS (Sigma-Aldrich) for 24 h. Concurrently cells were either incubated with untreated or Endo H-treated NST-GFP (20 μg/ml) for 24 h. Chemotaxis of activated BM-DCs was quantified using transwell migration assays. BM-DCs (2×10^5^ cells in 300 μl VLE-RPMI, Biochrom/0.5 % FCS, Sigma-Aldrich) were placed to the upper compartment of uncoated polycarbonat filters (Costar, Corning, 5 μm pore size) and subsequently incubated at 37 °C/5% CO_2_ to adhere. After 1 h 200 ng/ml CCL19 (PeproTech) was added to VLE-RPMI/0.5% FCS in the lower compartment. Control assays were performed without chemokine. Transmigrated cells were counted after incubation for 4 h at 37 °C/5% CO_2_.

### *In vitro* T cell activation assays

BM-DCs were pre-incubated with 20 μg/ml untreated β-gal, Endo H-treated β-gal, 1 μg/ml LPS or medium only. After intense washing, cells were pulsed with 125 nM OVA_257-264_or 800 nM OVA_323-339_ and incubated with OT I or OT II T cells. T cell activation was determined by measuring the amount of IL-2 in the supernatant after 18 h by ELISA.

To analyze T cell proliferation, OT I and OT II T cells were stained with 1 μM carboxyfluorescein succinimidyl ester (CFSE) before addition to the DCs. T cell proliferation was measured by flow cytometry after 3 days. Expansion index was calculated as previously described [43].

### *In vivo* T cell activation assays

Mice were immunized subcutaneously into the inguinal area at day -13 and day -6 with 50 μg glycosylated or de-glycosylated β-gal, NST-GFP-S8L, 4xNST-GFP-S8L, β-gal-S8L, NST-GFP-I17R or NST-GFP-TRP2 and 50 ng LPS per injection. At day -1, mice were injected intravenously with a mixture of 5×10^6^ control cells and 5×10^6^ target cells. As control cells, C57BL/6J splenocytes were labeled with a low concentration of CFSE (0,15 μM). As target cells, C57BL/6J splenocytes were pulsed with the β-gal_497-504_ (ICPMYARV), OVA_257-264_ (SIINFEKL) or TRP2_180-188_ (SVYDFFVWL) peptide (2 μM) and labeled with a high concentration of CFSE (1.5 μM). After another 18 h, inguinal lymph nodes were collected and the specific killing was determined by flow cytometry as described before [44]. To determine the secretion of cytokines, 10^6^ cells from draining lymph nodes were re-stimulated with 2 μM of the described peptides. After another 18 h, IFN γ, IL-4 or IL-6 concentrations in the supernatant were determined by ELISA.

### *In vivo* T cell proliferation

Mice were injected with 2.10^6^ CFSE-labeled T cells as previously described [21]. After 1 day, mice were immunized subcutaneously with 50 μg glycosylated or de-glycosylated protein and 50 ng LPS per injection. After another 3 days, T cell proliferation was analyzed by flow cytometry.

### ELISA to determine IgG from blood

β-gal, NST-GFP-S8L, 4xNST-GFP-S8L, β-gal-S8L or NST-GFP-TRP2 (25 μg/ml) were coated to an ELISA plate at 4 °C for 18 h. Afterwards, plates were washed, incubated with serum from immunized mice (diluted 1/64 in PBS) for 2 h. β-gal- or GFP-specific antibodies (whole IgG) were detected with an HRP-conjugated whole IgG-specific antibody. Amounts of IgG were monitored after addition of 1 mg/ml 2,2'-azino-bis(3-ethylbenzothiazoline-6-sulphonic acid) by colorimetry at 405 nm. Results were normalized to sera from naive mice.

### Statistical analysis

Statistical significance was determined by one way ANOVA. P values were calculated using Tukey HSD post-hoc analysis.

## SUPPLEMENTARY MATERIALS FIGURES


