# Supplementary material for: N-glycosylation converts non-glycoproteins into mannose receptor ligands and reveals antigen-specific T cell responses in vivo
Source: Oncotarget. 2016 Dec 28;8(4):6857–72. doi: 10.18632/oncotarget.14314 (PMC5351675; doi:10.18632/oncotarget.14314)
Supplement: Supplementary file 1 [file oncotarget-08-6857-s001.pdf]

N-glycosylation converts non-glycoproteins into mannose receptor ligands and reveals antigen-specific T cell responses *in vivo*

SUPPLEMENTARY FIGURES

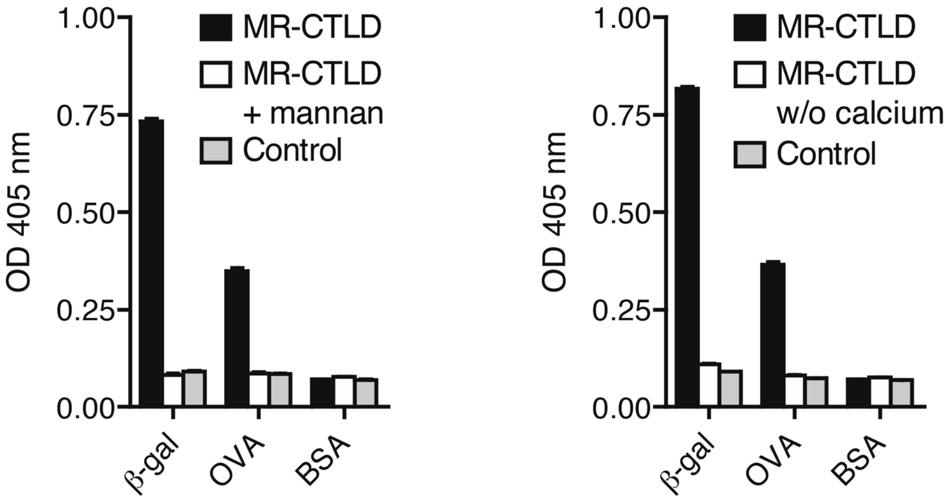

Supplementary Figure 1: Binding of MR-CTLD to β-gal and OVA is dependent on its C-type lectin activity. Binding of MR-CTLD or isotype control to β-gal, OVA or BSA in the presence of 3 mg/ml mannan (left) or in the absence of Ca^{2+} (right). Binding was determined by ELISA. Graphs show mean values ± SEM. All graphs depict representative examples of at least 3 independent experiments

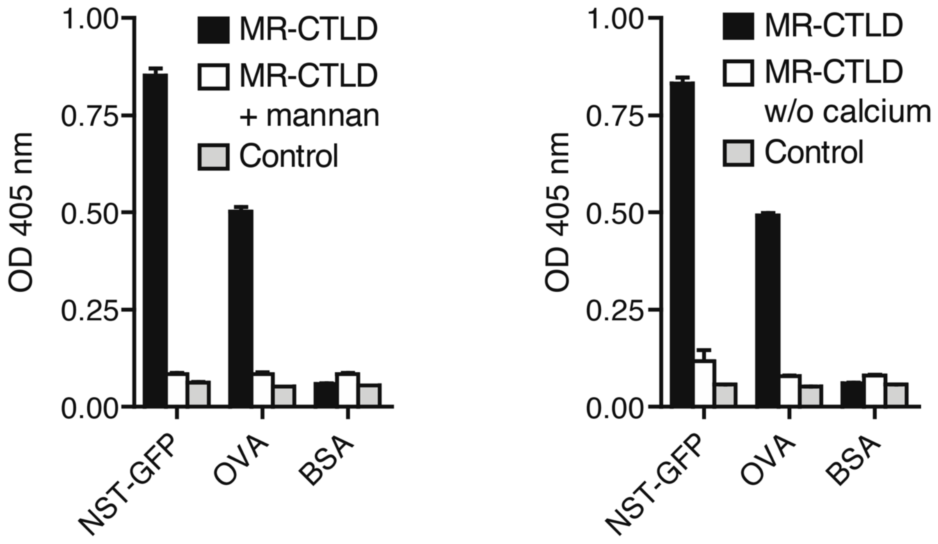

Supplementary Figure 2: Binding of MR-CTLD to GFP is dependent on its C-type lectin activity. Binding of MR-CTLD or isotype control to NST-GFP, OVA or BSA in the presence of 3 mg/ml mannan (left) or in the absence of Ca^{2+} (right). Binding was determined by ELISA. Graphs show mean values ± SEM. All graphs depict representative examples of at least 3 independent experiments.

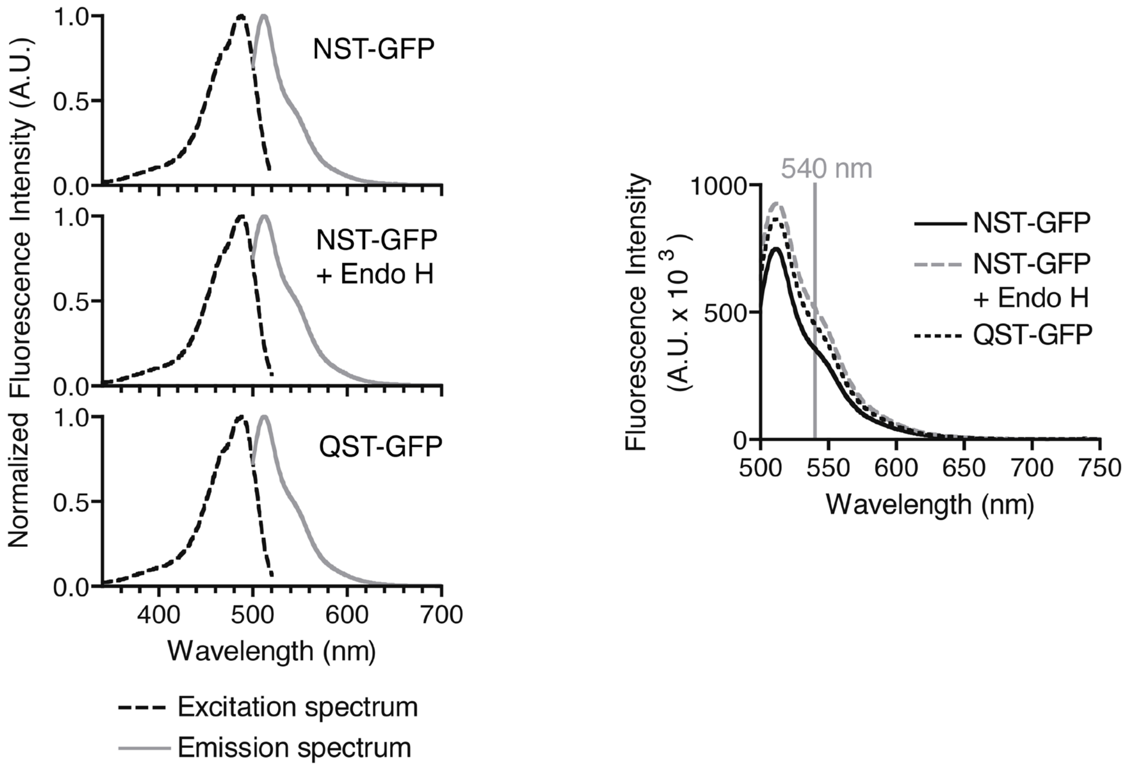

Supplementary Figure 3: Excitation and emission spectra of glycosylated or deglycosylated NST-GFP and QST-GFP. Excitation and emission spectra of purified NST-GFP and QST-GFP and of deglycosylated NST-GFP monitored by fluorescence spectroscopy.

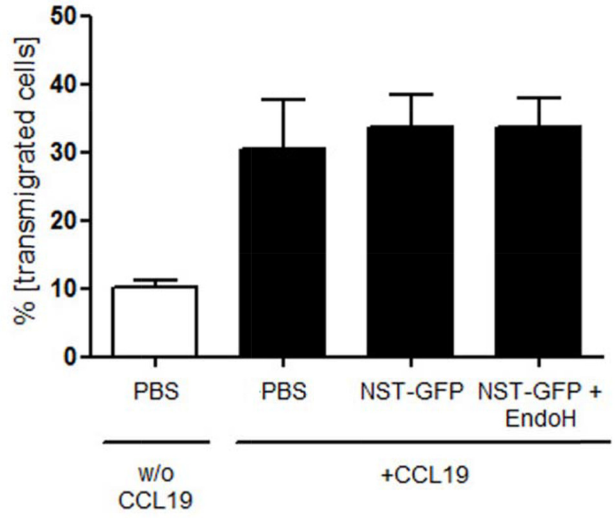

Supplementary Figure 4: Influence of the addition of N-glycosylated proteins to DC migration. BM-DCs were treated with untreated or EndoH-treated NST-GFP for 24h. Afterwards chemotaxis of DC towards CCL19 was determined using a transwell migration assay.
